# Identification of novel *CDH23* variants linked to hearing loss in a Chinese family: A case report

**DOI:** 10.1097/MD.0000000000039360

**Published:** 2024-09-13

**Authors:** Jing Sun, Dawei Ren, Meiheng Gong, Xinyi Guo, Yan Zhang, Bo Du

**Affiliations:** a Department of Otolaryngology Head and Neck Surgery, The First Hospital of Jilin University, Changchun, Jilin, China.

**Keywords:** *CDH23*, hearing loss, heredity, Mutation, NSHL

## Abstract

**Rationale:**

Deafness is associated with both environmental and genetic factors, with hereditary deafness often caused by mutations in deafness-related genes. Identifying and analyzing deafness-related genes will aid in early diagnosis and pave the way for treating inherited deafness through gene therapy in the future.

**Patient concerns:**

A 15-month-old girl underwent audiological examination at the outpatient clinic of the hospital due to hearing loss and her brother was diagnosed with profound bilateral sensorineural hearing loss at the age of 3.

**Diagnoses:**

The diagnosis was determined as extremely severe sensorineural hearing loss caused by genetic factors.

**Interventions:**

Clinical data of the patient were collected, and peripheral blood samples were obtained from both the patient and her family members for DNA extraction and sequencing.

**Outcomes:**

By utilizing targeted capture next-generation sequencing to further screen for deafness-related genes, 2 novel variants in *CDH23* were identified as the causative factors for the patient's deafness.

**Lessons:**

This study identified 2 novel heterozygous mutations in a Chinese family. Both the proband and her sibling have non-syndromic hearing loss (NSHL) and carry distinct heterozygous mutations of *cadherin-like 23* (*CDH23*). One mutation, *CDH23:c.2651 A>G*, originated from their mother and paternal family, affecting the exon23 domain of *CDH23*. The other mutation, *CDH23:c.2113 G>T*, was inherited from their paternal grandmother, impacting the exon19 domain of *CDH23*. These 2 novel mutations likely cause NSHL by affecting protein function. This finding suggests that identifying 2 novel mutations in *CDH23* contributes to the genetic basis of NSHL.

## 1. Introduction

Congenital hearing loss is among the most prevalent genetic disorders in children.^[1]^ Various factors contribute to congenital hearing loss, with growing recognition of genetic influences.^[2]^ Genetic factors are causative in many instances of hearing loss, with mutations impacting auditory pathway components.^[1]^ In the context of hereditary hearing loss, genetic testing constitutes an essential diagnostic step.^[3]^ A study found that approximately 1 in 1000 individuals in the United States are born deaf or experience significant hearing loss in early childhood.^[4]^ The presence of hundreds of mutated genes underscores the significant genetic heterogeneity of congenital hearing loss. Additionally, mutations associated with deafness may manifest diverse phenotypes.^[5]^

Over 50% of deaf children are estimated to have a genetic predisposition, with 70% of inherited deafness being non-syndromic.^[6]^ Various non-syndromic recessive genes are associated with gradual sensorineural hearing loss. In some cases, children may pass a hearing test shortly after birth but experience a progressive loss over time.^[7]^ There is genetic heterogeneity in hearing loss, with around 152 genes related to deafness being associated with non-syndromic hearing loss (NSHL).^[8]^ The prevalence of NSHL is increasing, with deafness occurring in 2.7‰ of childhood NSHL patients and 3.5‰ of adolescent patients.^[9]^

Mutations in autosomal genes such as *TMPRSS3*, *TMC1*, *USHIC*, *CDH23*, and *TMIE* are recognized as causes of hereditary hearing loss. In another study, mutations in these 5 genes accounted for 10% of autosomal recessive non-syndromic hearing loss observed in the investigated families.^[10]^ Various studies have reported that *cadherin-like 23* *(*CDH23**) can cause a spectrum of NSHL phenotypes, ranging from severe congenital hearing loss to progressive high-frequency hearing loss that develops later in life.^[11]^ Within the cadherin superfamily, the *CDH23* gene is expressed in both inner and outer hair cells in the cochlea and encodes calcium-dependent cell-cell adhesion glycoproteins.^[11]^ The encoded protein cadherin 23 is located at the top of tip links and plays a crucial role in preserving the function of hair cells.^[12]^ Mutations in *CDH23* disrupt tip link formation and bundle morphology, leading to the inability of mechanotransduction channels to maintain their open state during rest periods.^[13]^ Therefore, mutations in *CDH23* may contribute to deafness.

Gene capture and Sanger sequencing were employed in this study to detect genes associated with hearing loss in a family of children. The proband diagnosed with NSHL was found to have 2 novel heterozygous mutations *(CDH23: c.2651 A>G* and *CDH23: c.2113 G>T*). Additionally, the results indicate that the proband’s *CDH23: c.2651 A>G* mutation was inherited from the mother, while the *CDH23: c.2113 G>T* mutation in the proband was traced back to the grandmother. This study reports the identification of 2 novel mutations *(CDH23: c.2651 A>G* and *CDH23: c.2113 G>T*) in *CDH23* within a Chinese family, previously undocumented. Furthermore, this study contributes to the understanding that NSHL can result from *CDH23* mutations.

## 2. Materials and methods

### 2.1. Study subjects

**Figure 1.**
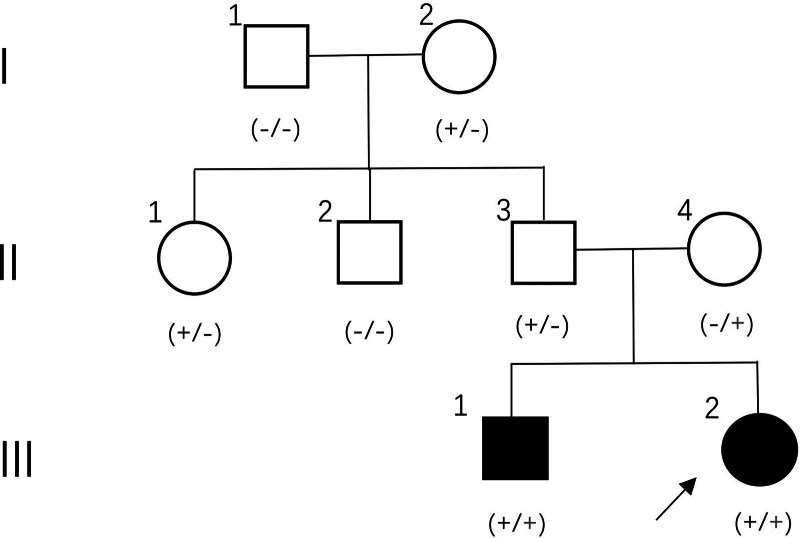
Pedigree of the family with hearing loss due to a pathogenic variant. Affected individuals are depicted in black, with the proband indicated by an arrow. The black “+” represents the *c.2113 G>T (p.Gly705Cys)* mutation, while the red “+” represents the *c.2651 A>G (p.Asn884Ser)* mutation.

The study received approval from the Ethics Committee of the First Hospital of Jilin University (Changchun, China), and all participants provided written informed consent before enrollment. The study included a patient (III:1) with severe sensorineural hearing loss and her paternal grandparents (I:1 and I:2), paternal aunt (II:1), paternal uncle (II:2), parents (II:3 and II:4), and sibling (III:1). The proband (III:2) (Fig. 1), a 15-month-old girl, was diagnosed with severe sensorineural hearing loss. The proband’s brother was diagnosed with profound bilateral sensorineural hearing loss at the age of 3. Clinical information and peripheral blood samples were collected for DNA extraction and sequencing.

### 2.2. *DNA* s*ample* c*ollection*

Peripheral blood samples (5 mL) were collected from each individual for DNA analysis. Genomic DNA extraction was performed using a TIANamp Blood DNA Kit (Tiangen Biotech, Beijing, China). The genomic DNA products, with a concentration of 100 to 150 ng/μL and an OD260/OD280 ratio of 1.7 to 1.9, were purified following the manufacturer’s instructions and stored at −20 °C.

### 2.3. Targeted deafness gene capturing and DNA sequencing

The SureSelect Human All Exon V6 kit was used to capture the target gene using the whole-exon liquid-phase hybridization capture method. Sequencing was conducted on an Illumina next-generation sequencing platform.

### 2.4. Confirmation by Sanger sequencing

The following primers were used to verify the mutation through PCR-based Sanger Sequencing: For *CDH23* *exon 19*, forward 5′-*TTGGGATGGAGGGCTCTGAAT*-3′ and reverse 5′-*CTGGGCTGGTAGGAATGAGATG*-3′; for *CDH23* exon 23, forward 5′-*CAAGAGCAACGATTGAGCCG*-3′ and reverse 5′-*GTGGAGGACCAGGGTACTTG*-3′. PCR amplification was conducted with the following conditions: 95 °C for 15 minutes; followed by 32 cycles of denaturation at 96 °C for 1 minute, annealing at 94 °C for 30 seconds, extension at 55 °C for 30 seconds, and final extension at 72 °C for 5 minutes. Purified PCR products with sizes of 295 and 350 bp were subjected to Sanger sequencing (Comate Bioscience Co., Ltd).

### 2.5. Data analysis

Sequence data were analyzed and aligned with the *CDH23* reference sequence from the National Center for Biotechnology Information (NCBI) using DNA Star 5.0 software. Additionally, the novelty of variants found in this study was assessed using the NCBI dbSNP database and the 1000 Genomes Project database (http://www.1000genomes.org/) as references.

## 3. Results

### 3.1. Patient clinicopathological features

**Figure 2.**
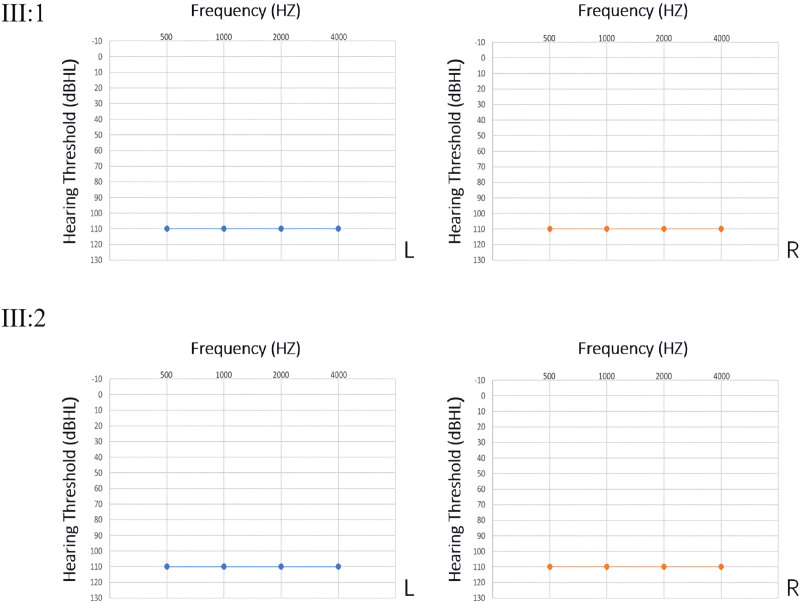
Pure-tone audiometry results of proband and her sibling.

The proband (III:2), a 15-month-old female, had bilateral severe sensorineural hearing loss, while her brother (III:1), aged 3, also had bilateral hearing loss. Audio steady-state response (ASSR) revealed profound sensorineural hearing loss at all frequencies for the proband (Fig. 2). The proband’s paternal grandparents, aunt, uncle, and parents had normal hearing and showed no impairments in vestibular, movement, or visual functions.

### 3.2. Conservation analysis of the mutation sites

**Figure 3.**
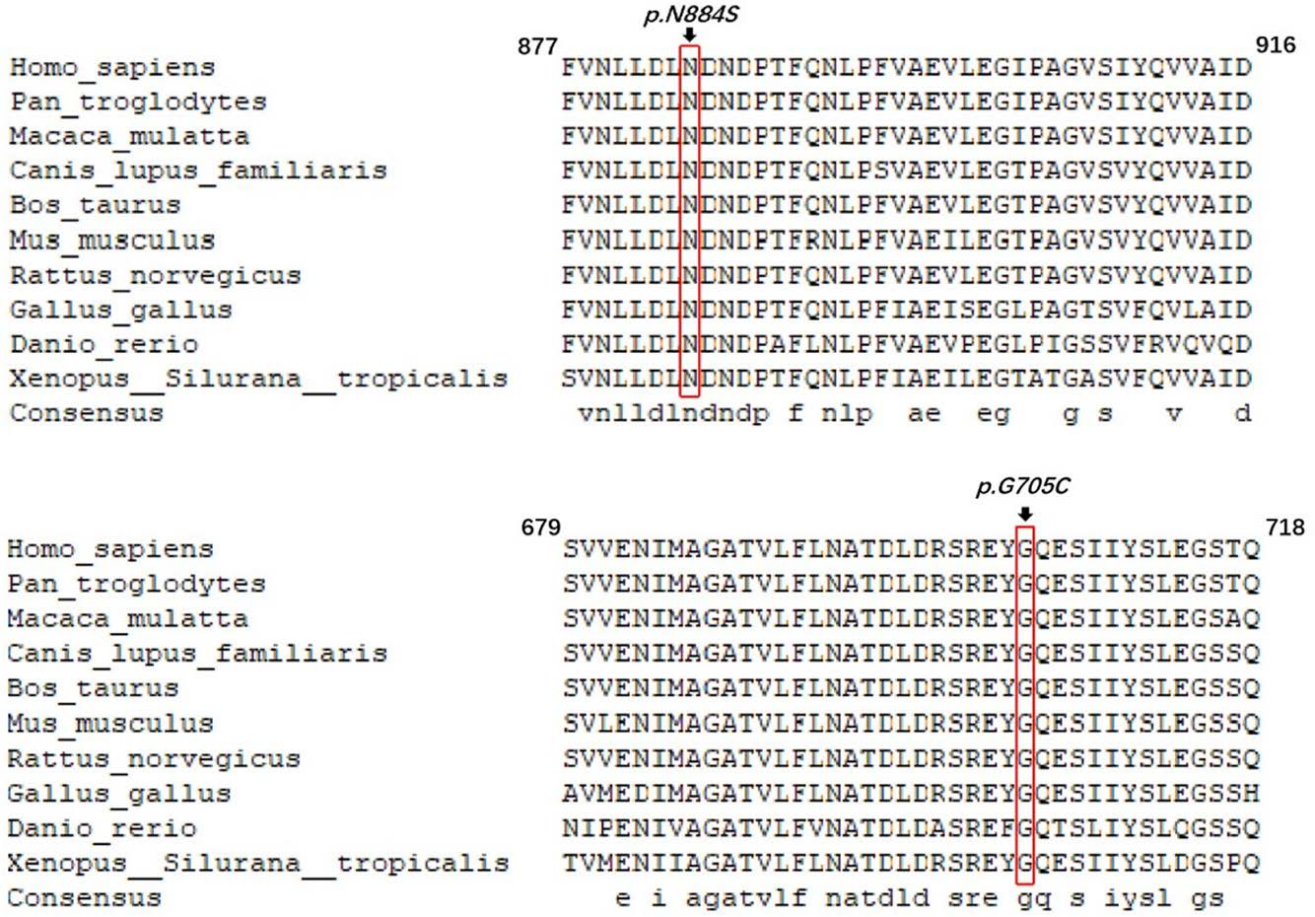
Protein alignment of *CDH23* in different species, which shows the conservation of residue *p. Asn884Ser* and *p. Gly705Cys.*

One mutation identified in this family results in an amino acid change from glycine to cysteine *(p.Gly705Cys*), while another mutation results in an amino acid change from asparagine to serine *(p.Asn884Ser*). Information about *CDH23* was obtained from NCBI, and the gene was analyzed using DNAMAN software (Fig. 3). Glycine and asparagine are highly conserved amino acids in vertebrate species.

### 3.3. Bioinformatics analysis of the mutations

Both SIFT and PROVEAN predictions were utilized to assess the effects of the 2 identified variants. The results indicated that the variant *CDH23 (NM_022124*) *exon19* *c.G2113T p.Gly705Cys* is associated with the risk of hearing loss. The PolyPhen prediction score suggests that both identified variants can cause damage. The MutationTaster prediction score also indicates that both variants are potentially damaging, implying a negative impact on protein function. Overall, the predictions for the 2 mutated genes suggest that they may result in profound sensorineural hearing loss (Table 1).

**Table 1:** Bioinformatics analysis of the mutations.

Mutation	SIFT		PolyPhen2		MutationTaster		FATHMM		PROVEAN	
	Score	Prediction	Score	Prediction	Score	Prediction	Score	Prediction	Score	Prediction
CDH23 NM_022124 exon23 c.A2651G p.N884S	.	.	0.987	Probably damaging	1	Disease causing	.	.	.	.
CDH23	0.01	Deleterious	1	Probably damaging	1	Disease causing	.	.	−3.22	Deleterious
NM_022124										
exon19										
c.G2113T										
p.G705C										

PolyPhen Prediction Score: ≤0.5 (benign); >0.5 (probably damaging). MutationTaster Prediction: polymorphism or disease-causing. SIFT Prediction (cutoff = 0.05): tolerated or damaging. PROVEAN Prediction (cutoff = −2.5): deleterious or neutral. FATHMM Prediction (weighted): tolerated or damaging.

### 3.4. Genetic analysis

**Figure 4.**
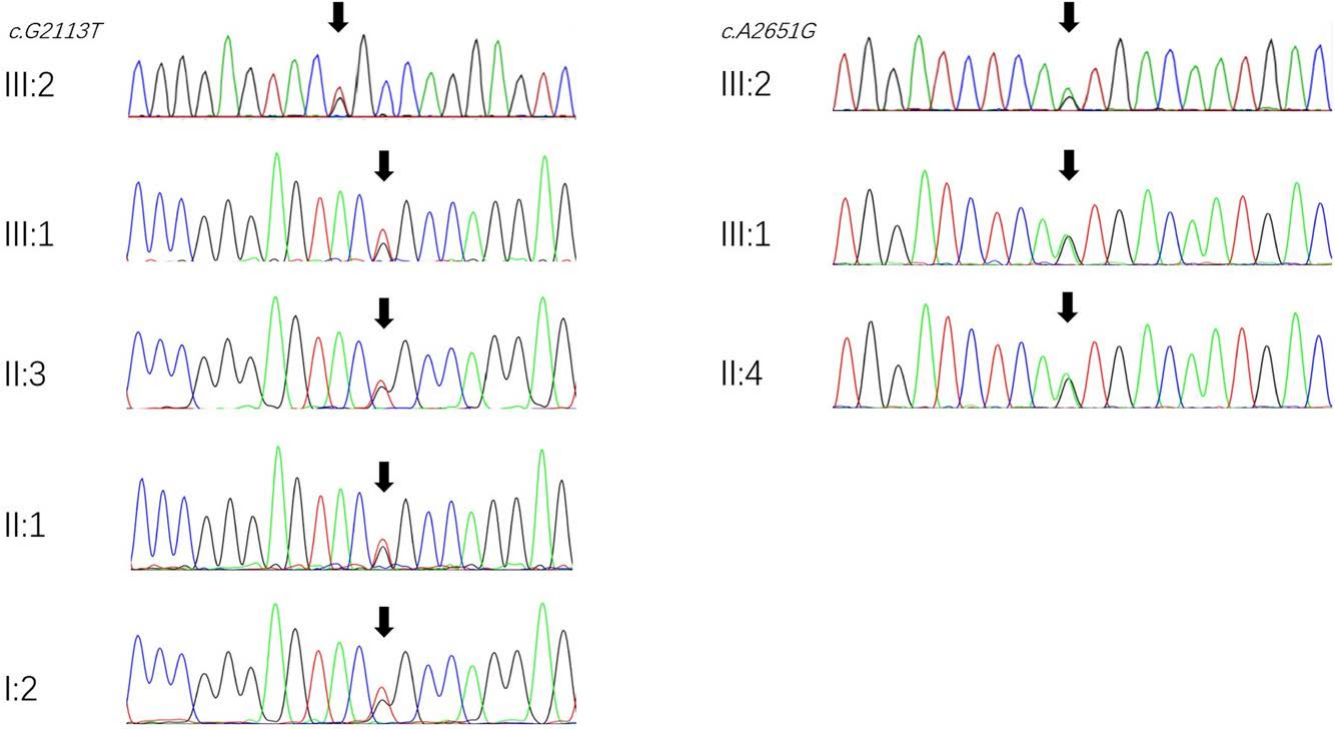
Sanger sequencing result of *c.2113 G>T* *(p. Gly705Cys*) and *c.2651 A>G* *(p. Asn884Ser*) variants in *CDH23*.

The study examined 9 deafness-causing hotspots in the 4 most common genes: *GJB2* *(c.35delG*, *c.176del16*, *c.235delC*, and *c.299delAT*), *GJB3* *(c.538C>T*), *SLC26A4* *(c. IVS7-2A>G* and *c.2168A>G*), and mitochondrial *MT-RNR1* *(c.1494C>T* and *c.1555A>G*). Multiple PCR analyses were conducted, but no mutations were detected in these hotspots. The screening for deafness-causing genes then continued with targeted capture next-generation sequencing, revealing 2 novel variants of *CDH23* responsible for deafness in this patient. Experimental data showed that 2 heterozygous mutations of *CDH23* originated from their relatives (Fig. 4). One of the mutations, *CDH23: c.2651 A>G*, from the proband’s mother, was detected in exon 23 of *CDH23*, while another, *CDH23: c.2113 G>T*, from the proband’s maternal grandmother, was detected in exon 19 of *CDH23*. Notably, the proband’s paternal grandmother, paternal aunt, father, and brother all carry the second mutation. Therefore, these 2 novel missense mutations of *CDH23* may cause profound sensorineural hearing loss.

## 4. Discussion

*CDH23*, a member of the supramolecular cadherin family, has garnered increasing attention in recent years due to its complex structure and biological function. Variations in the extracellular regions have led to the recognition of the great potential of this superfamily.^[14]^ Recent studies report that 1 of the 2 members of the cadherin superfamily constitutes a part of the mechanical transduction mechanism of sensory hair cells in the inner ear of vertebrates. These studies suggest that some of the extracellular filaments are formed by *CDH23* and protocadherin 15 *(PCDH15*), connecting the kinocilium of the hair cell and stereocilia into the bundle.^[15]^ Mutations in the genes responsible for *PCDH15* and *CDH23* have been found to cause hearing impairment and balance disorders in both mice and humans.^[16]^ *CDH23*-related hearing loss is associated with its role in the tip links of the inner ear hair cells. The tip links are extracellular filaments considered as the gateway to mechanical transduction channels. *CDH23* enables humans to hear and maintain balance by transducing the mechanical forces arising from sound waves and head movements.^[17,18]^ Mutations in *CDH23* disrupt tip link formation and bundle morphology, resulting in the inability of mechanotransduction channels to maintain their open state during periods of rest.^[13]^ Thus, mutations of *CDH23* could cause hearing loss.^[15]^

In this study, 9 known hotspot mutations associated with the 4 most common deafness genes were investigated: *GJB2 c.35delG*, *c.176del16*, *c.235delC*, and *c.299delAT*; *GJB3 c.538C>T*; *SLC26A4 c. IVS7-2A>G* and *c.2168A>G*; and mitochondrial *MT-RNR1 c.1494C>T* and c*.1555A>G*. However, the analysis yielded negative results. Nonetheless, 2 novel missense mutations of *CDH23* were identified, suggesting a potential association with hearing loss. Both mutations occurring in a highly conserved region of the protein (Fig. 3) suggest that the affected amino acids play a pivotal role in maintaining protein functionality. Missense mutations within conserved regions often significantly impact protein function, potentially resulting in hearing loss or other auditory impairments. Missense mutations in genes, including *CDH23*, may represent a common etiology of deafness,^[19]^ depending on the mutational site within the protein. Gene sequencing results suggest that 2 mutations of *CDH23* originated from the proband’s relatives.

**Figure 5.**
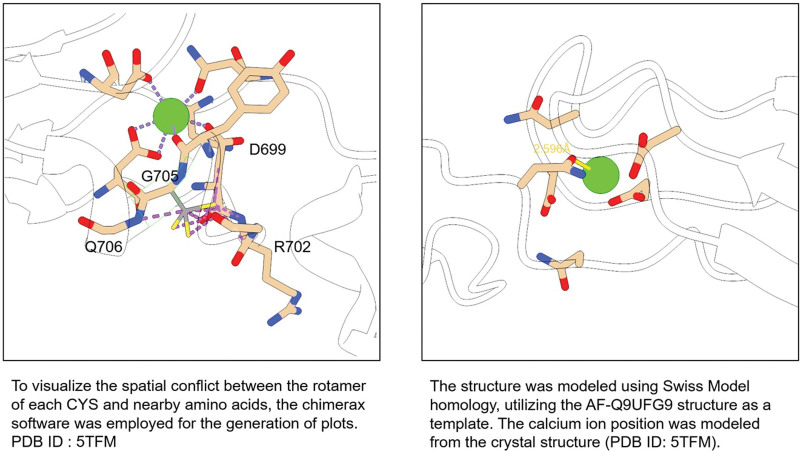
The structures of *c.2113 G>T* *(p.Gly705Cys*) and *c.2651 A>G* *(p. Asn884Ser*) variants in *CDH23*.

In our study, the mutations result in a glycine-to-cysteine substitution and a serine-to-asparagine substitution. As shown in Figure 5, significant alterations in protein structure occur following these amino acid substitutions. The protein structure was constructed using the PyMOL Molecular Graphics System (https://pymol.org/2/). *G705*, located at the junction of the loop and the helix, is essential for forming the loop that constitutes the calcium ion binding site. The *G705C* mutation affects the flexibility of the backbone, disrupting loop formation. Additionally, it clashes with neighboring residues *Q706*, *R702*, and *D699*, particularly *D699*, directly affecting the coordination between *D699* and calcium ions. Ultimately, these effects compromise calcium ion binding. *N884*, located between the 2 cadherin domains, is responsible for binding calcium ions. Mutation of *N884* to serine results in a shorter side chain and a greater distance from the calcium ions, impairing calcium ion binding. In conclusion, both mutations may contribute to hearing impairment by interfering with calcium ion binding.

Comprehensive genetic screening not only affords clinicians a high diagnostic yield but also furnishes valuable insights into the identification of subclinical or presymptomatic phenotypes.^[20]^ The Human Gene Mutation Database (HGMD) has documented at least 492 distinct mutations in *CDH23*.^[21]^ Other related studies discovered compound heterozygous missense mutations [*p.(Asp918Asn*); *p.(Val1670Asp*)] in *CDH23* through exome sequencing. These studies revealed that both the *p.(Asp918Asn*) and *p.(Val1670Asp*) mutations identified in the patients affected conserved *CDH23* amino acids and were predicted to have serious detrimental effects by multiple in silico tools.^[22]^ Another study demonstrated that mutations in *CDH23* led to defects in purine metabolism, resulting in insufficient ATP, which is essential for the normal function of hair cells, and consequently led to hearing loss.^[23]^ This provides supporting evidence to our study, suggesting that these 2 novel mutations found could result in hearing loss.

In pediatric populations with sensorineural hearing loss, the absence of timely therapeutic interventions can lead to substantial deficits in linguistic competence, cognitive performance, and scholastic achievement, consequently compromising long-term developmental outcomes and life prospects.^[24]^ Preliminary animal research indicates that prenatal gene therapy effectively addresses genetic hearing impairment, exhibiting minimal germline alterations and no observed adverse off-target consequences.^[25]^ In the recent study by Sun et al, it is demonstrated that in utero gene therapy (IUGT) holds substantial promise for the treatment of irreversible pathological manifestations of hereditary hearing loss that commence prenatally or shortly after birth.^[26]^ Given that *CDH23* is a prevalent gene associated with hereditary deafness, its screening in clinical practice is imperative. Future research may explore gene therapeutic interventions targeting pathogenic mutations within this gene, potentially leading to significant improvements in pediatric hearing, a reduction in the necessity for cochlear implantation, and a substantial decrease in surgical risks and the financial burden on patient families.

## 5. Conclusions

In this study, we report 2 novel variants of *CDH23* in the Chinese population, providing a new example of severe sensorineural hearing loss caused by *CDH23* mutations. Both mutations may contribute to hearing impairment by interfering with the binding of calcium ions. This study has significantly expanded the spectrum of mutations associated with *CDH23*-linked hearing loss.

## Acknowledgments

We are sincerely grateful to Ping Wang for her comments of the manuscript.

## Author contributions

**Data curation:** Jing Sun.

**Writing – original draft:** Jing Sun.

**Investigation:** Dawei Ren.

**Validation:** Meiheng Gong, Xinyi Guo.

**Software:** Xinyi Guo.

**Writing – review & editing:** Xinyi Guo, Yan Zhang, Bo Du.

**Conceptualization:** Bo Du.
